# Cellulose acetate nanoparticles: impacts of concentration and exposure time on the biochemistry and hematology of juvenile *Colossoma macropomum*

**DOI:** 10.1007/s10646-026-03174-w

**Published:** 2026-10-23

**Authors:** Camila Gomes de Oliveira, Túlio Pacheco Boaventura, Fábio Aremil Costa dos Santos, André de Sena Souza, Sidney dos Santos Silva, Imaculada de Morais Carvalho Ananias, Camila Silva Brey Gil, Ronald Kennedy Luz, Rodrigo Lambert Oréfice

**Affiliations:** 1 Department of Metallurgical and Materials Engineering, Federal University of Minas Gerais (UFMG), Av. Antônio Carlos, 6627 - Campus da UFMG, Pampulha, Belo Horizonte, MG Brazil; 2 Departamento de Zootecnia, Laboratório de Aquacultura, Universidade Federal de Minas Gerais, Avenida Antônio Carlos, n° 6627, Belo Horizonte, CEP 30161- 970 MG Brazil; 3 Department of Engineering, Federal University of Lavras, PO Box 3037, Lavras, 372000-900 MG Brazil

**Keywords:** Tambaqui, Cellulose acetate, Stress, Hematological parameters, Physiological recovery

## Abstract

This study evaluated the physiological impacts of acute-exposure of tambaqui (*Colossoma macropomum*) to different concentrations of cellulose acetate nanoparticles at different sampling times. A total of 120 juvenile *C. macropomum* (38.91 ± 5.57 g and 13.76 ± 0.74 cm in total length) were distributed in 12 tanks of 150 L, in a recirculating aquaculture system (RAS). The juveniles were kept in water containing cellulose acetate nanoparticles (CANs) at four different concentrations (treatments): 0 mg L^−^¹, 0.01 mg L^−^¹, 0.10 mg L^−^¹ and 1.00 mg L^−^¹. Blood samples were collected after 1 and 24 h of exposure. The juveniles were then relocated to a tank with clean water, where they remained for another 24 h, after which a new blood sample was collected (24 h). Hemoglobin, serum protein and globulin were reduced after 1 h of exposure for the group exposed to 1.00 mg L^−^¹ of CANs. Glucose levels increased after 24 h for the groups exposed to 0.10 and 1.00 mg L^−^¹. Urea increased after 1 h for the groups exposed to 0.10 and 1.00 mg L^−^¹. Triglycerides and cholesterol were reduced both after 1 h and 24 h of exposure for the 1.00 mg L^−^¹ group, returning to baseline levels at 24 h. The activities of aspartate aminotransferase (AST) and alanine aminotransferase were altered after 24 h. Albumin was lower after 1 h and 24 h compared to after 24 h for the 1.00 mg L^−^¹ group. Exposure of juvenile *C. macropomum* to CANs caused transient physiological changes, with partial recovery being observed after transfer to clean water.

**Supplementary information:**

The online version contains supplementary material available at https://doi.org/10.1007/s10646-026-03174-w.

## Introduction

Human population growth and increased industrial activities have driven an increasing global demand for plastics, resulting in significant growth in its production. Global plastic production was approximately 2 million tons in 1950, while in 2022 this volume was estimated at 400.3 million tons (Pilapitiya and Ratnayake 2024). This expansion is attributed to the unique properties of plastics, such as low cost, light weight, versatility, and high durability, which make them very important to several sectors (Babaremu et al. 2023). However, these same characteristics that favor the use of these materials also represent major environmental challenges (Adamovsky et al. 2021). Freshwater ecosystems are particularly affected, as they act as receptors for pollutants from river basins (Azevedo-Santos et al. 2021; Pilapitiya and Ratnayake et al. 2024).

Cellulose acetate (CA) is one of the most commercially important polymers derived from cellulose. This material has a wide range of applications, such as in the textile industry and as films for biomedical applications; it is also widely used to manufacture cigarette filters (Puls et al. 2011; Konwarh et al. 2013). Cellulose acetate is one of the most common plastic contaminants found in aquatic environments, especially marine environments (Munari et al. 2016; Rebischung et al. 2018). It is considered resistant to biodegradation (Dieng et al. 2013; Torkashvand et al. 2020), but can be classified as photodegradable, meaning that under the right conditions it can be degraded by the action of ultraviolet radiation, fragmenting into smaller particles such as micro- and nanoplastics, which can remain in the aquatic environment for up to 15 years (Marah and Novotny 2011; Dieng et al. 2013; Torkashvand et al. 2020). Cellulose acetate nanoparticles (CANs) have also been used as carriers of agricultural inputs and can flow directly into water bodies (Callaghan et al. 2023).

Plastic particles can be classified according to their size into the following: macroplastics, which are larger than 5 mm; microplastics, which are between 0.1 and 5 mm; and nanoplastics, which are smaller than 1000 nm for some authors (Baptista Neto et al. 2020; Strafella et al. 2020; Lee et al. 2021; Ramkumar et al. 2022) and smaller than 100 nm for others (Koelmans et al. 2015). Nanoplastics can cause physiological impacts to fish and, consequently, affect essential biological processes and survival (Liang et al. 2023). Aquatic organisms can be exposed to plastic particles through gill respiration and gastrointestinal ingestion (Hayati et al. 2023). After exposure, nanoparticles can cross the epithelium through mechanisms such as endocytosis, reaching the circulatory system (Hu and Palić 2020; Lall and Kaushik 2021). Among the various impacts that nanoplastics can have is the accumulation in vital organs, such as the intestine, liver and brain, as they are able to cross cell membranes (Sarasamma et al. 2020; Wang et al. 2022). Once in circulation, they can induce adverse cellular responses, compromising tissue integrity and functionality (Joksimovic et al. 2022), triggering metabolic disorders, changes in lipid metabolism, inflammatory responses, oxidative imbalances, liver dysfunction, and reproductive interference (Ma et al. 2020; Yu et al. 2022; Wang et al. 2023; Zang et al. 2024). Consequently, fish homeostasis may be affected, activating compensatory mechanisms detectable in blood parameters (Guerrera et al. 2021). In addition, particles can contribute to vascular obstructions by interacting with circulating components such as erythrocytes, albumin, and globulins (Jo et al. 2025). Thus, blood parameters can act as integrated biomarkers of the systemic effects associated with exposure to nanoplastics.

Tambaqui, *Colossoma macropomum* (family Characidae), is a native freshwater fish species of the Amazon basin (Brian et al. 2003). Andrade et al. (2019) reported microplastics in the stomach contents of several species, representing different trophic guilds (herbivores, omnivores and carnivores), in the same region, evidencing negative impacts on aquatic fauna. Several studies have demonstrated the effects of nanoplastics on aquatic organisms, especially polyethylene, polystyrene, and polypropylene (Hamed et al. 2022; Hossinian et al. 2025; Kim and Kim 2025). However, research involving CANs is still scarce. The studies found on the effects of cellulose acetate on aquatic organisms involve microplastics derived from cigarette butts (Belzagui et al., 2021; Cho et al. 2025), and research on the potential effects associated with exposure to particles at the nanoscale remains limited. Cigarette butts are among the most commonly found plastic pollutants in the environment (Silva et al. 2018; Dobaradaran et al. 2021). Studies have investigated the effects of used cigarette butts and their leachates, which may contain various contaminants absorbed from smoke (Osuala et al. 2017; Soleimani et al. 2023; Banaee et al. 2024). Therefore, it is difficult to distinguish the effects specifically attributable to cellulose acetate from those caused by other contaminants associated with cigarette butts, which can have various effects on fish (Osuala et al. 2017). Consequently, information on the effects of CANs alone remains limited. Further studies are needed to evaluate the effects of this polymer at the nanoscale on aquatic organisms.

Thus, this study aimed to investigate the effects of acute-exposure to different concentrations of cellulose acetate nanoparticles for sampling times on the physiological responses of juvenile *C. macropomum* related to intoxication and stress.

## Materials and methods

### Production and characterization of CANs

The nanoemulsion containing CANs used in the present study was produced by the emulsification solvent evaporation technique, based on the methodology proposed by Fodor-Kardos et al. (2020) and Lima et al. (2022), with some adaptations. Previously, unused cigarette filters (butts) from three different brands were separated from other components and used as raw material for the extraction of CA. The filters were analyzed using Fourier transform infrared spectroscopy (FTIR) on a Thermo Scientific Nicolet 6700 instrument equipped with an Attenuated Total Reflectance (ATR) accessory. Spectra were collected in absorption mode over the range of 650–4000 cm^−^¹, with a spectral resolution of 4 cm^−^¹ and 32 scans per sample, using a ZnSe crystal.

The filters were dissolved in dichloromethane (Synth, 99% purity) at a ratio of 1:50 (m/v), under stirring at 30 °C for two hours using a magnetic stirrer. The obtained solution was then mixed with 100 mL an aqueous solution of 2% (w/V) polyvinyl alcohol (PVA) in distilled water (Sigma-Aldrich, 99% CAS 9002-89-5) using a mechanical mixer (Ultra-Turrax, IKA T10) operating at 14,500 rpm for 10 min at 27 °C.

After initial emulsification, the system was subjected to nanoemulsification with an ultrasonic tip sonicator (Ultronique, Disruptor, Indaiatuba-SP), equipped with a 4 mm diameter probe. Sonication was performed in two 3-min cycles (with 1-min intervals between cycles) at 70% amplitude. The nanoemulsion was then kept under constant stirring at 40 °C for 24 h to promote evaporation of dichloromethane. The concentration of nanoparticles dispersed in water was determined by weighing the resulting solution after dichloromethane evaporation. After seven days, the resulting CANs were characterized by dynamic light scattering (DLS), zeta potential and atomic force microscopy analysis (AFM). Measurements of diameter (with DLS) and zeta potential were performed on samples of CANs diluted at a concentration of 0.3 mg/mL. Both DLS and zeta potential tests were performed using ZetaPALS equipment (Brookhaven Instrument). Five measurements were performed for each of five independent samples, and the mean and standard deviation were calculated. The intensity distribution was extracted from the equipment software and used to calculate weighted mean diameter. Morphological characterization of the CANs was performed using a Shimadzu Model SPM 9600 atomic force microscope. The 0.3 mg/mL dilution was dropped onto glass slides, dried at room temperature and fixed in the sample holder. Images were acquired in non-contact mode at a scan rate of 0.8 Hz using a silicon microcantilever. ImageJ software was used to measure morphology parameters of at least 70 CANs.

### Experimental conditions and exposure to CANs

The experiment was conducted at the Laboratório de Aquicultura (LAQUA) of the Universidade Federal de Minas Gerais (UFMG), Belo Horizonte, Minas Gerais, Brazil, and approved by the Comitê de Ética em Pesquisa of CEUA, UFMG (Protocol 78/2025). The study was conducted and reported in accordance with the ARRIVE 2.0 guidelines.

One hundred and twenty juvenile *C. macropomum*, with an average weight of 38.91 ± 5.57 g and an average total length of 13.76 ± 0.74 cm, were randomly distributed into 12 150 L tanks (useful volume of 100 L), at a density of 10 juveniles/tank, in a recirculating aquaculture system (RAS) with constant aeration.

The juveniles were acclimatized for 15 days and fed a commercial extruded diet (Acqua: 32% crude protein, 6% ether extract, 6% crude fiber, 12% moisture and 13% mineral matter) once a day until apparent satiety. Water quality parameters were measured daily — temperature (28.35 ± 0.82 °C) and pH (8.29 ± 0.06) were measured using a portable pH meter (HANNA - HI 98107), dissolved oxygen (5.95 ± 0.27 mg L^− 1^) with an oximeter (HANNA-H1946) and total ammonia (0.96 ± 0.78 mg L^− 1^) using an colorimetric kit (Labcon Test, Alcon Pet, Camboriú, SC, Brazil). The photoperiod was 12 L:12D.

The impact of CANs was assessed by interrupting the RAS before exposure throughout the 24-hour blood collection period, with continuous and abundant aeration. The airflow was positioned at the bottom of the tank to prevent particle sedimentation, and no water renewal was performed in order to avoid changes in nanoparticle concentration.

Juveniles were exposed to one of four concentrations of CANs: 0 mg L^−^¹ (control), 0.01 mg L^−^¹, 0.10 mg L^−^¹ and 1.00 mg L^−^¹. The concentrations were acquired by weighing CANs on a precision scale (NET-AD330 Scales) and dilution in the amount of water required by the respective treatments. The lowest concentrations of CANs used in this study were determined based on previous research on the concentration of microplastics in water and to encompass environmentally relevant values. In addition to these concentrations, extreme scenarios were also considered, with higher levels to simulate the future impacts of increased concentrations of plastics in aquatic environments if plastic pollution continues to increase in the coming years, thus ensuring concentrations sufficient to induce significant physiological responses (Sendra et al. 2019; Al-Sid-Cheikh et al. 2018; Marana et al. 2022).

The experimental design consisted of four treatments corresponding to CAN concentrations of 0 (control), 0.01, 0.10 and 1.00 mg L^−^¹, with three replicates per treatment, totaling 12 experimental units (tanks). To assess the physiological effects of CAN exposure, three blood sampling times were established: after 1 h and 24 h of exposure acute-exposure and after a recovery period, when the juveniles were transferred to clean water and remained there for 24 h (24 h). Each tank was designated for only one sampling time. Therefore, the tank was considered the experimental unit (*n* = 3 tanks per CAN concentration), and the juveniles sampled in each tank were treated as subsamples. For statistical purposes, the average of the 10 fish per tank was used as a single observation for that experimental unit. This experimental approach was adopted to avoid potential physiological interference associated with repeated handling and blood sampling stress. Sampling times were selected to assess immediate physiological responses to acute-exposure to CANs, as well as possible changes in these responses over the course of the exposure period. The 24 h period was included to assess possible changes in physiological responses after the CANs were removed from the exposure system. The juveniles were fasted for 24 h before each collection.

Two blood samples were collected from each juvenile by puncture of the vertebral vein. The juveniles were captured individually in the tanks and restrained in a damp cloth while approximately 0.6 mL of blood was withdrawn, using a 1 mL syringe with a 25 mm x 0.7 mm heparinized needle, to which 10% heparin was subsequently added. Another 0.3 mL of blood was collected from the same animals using syringes without anticoagulant.

The samples with anticoagulant were used for hemoglobin evaluation using color reagent (Bioclin^®^, Belo Horizonte, MG, Brazil) (Tonks 1983), hematocrit using the microhematocrit method (Goldenfarb et al. 1971) and erythrocytes stained in Natt-Herrik solution followed by counting in a Neubauer chamber. Mean corpuscular volume (MCV), mean corpuscular hemoglobin (MCH) and mean corpuscular hemoglobin concentration (MCHC) were then calculated according to the formulations established by Wintrobe (1934). The remaining aliquots (with and without anticoagulant) were then centrifuged at 4000 rpm for 10 min to obtain plasma to determine glucose (catalog no. K310-2), cholesterol (catalog no. K306-2-VET), triglycerides (catalog no. K316-2), alanine aminotransferase (ALT; catalog no.K049-6) and aspartate aminotransferase (AST; catalog no.K048-6) activities, and serum to analyze serum protein (catalog no. K315-2), albumin (catalog no. K304-1) and globulin by difference. All analyses were performed using commercial kits from Bioclin^®^ (Quibasa Química Básica Ltda., Belo Horizonte, MG, Brazil) and reading with a semiautomatic analyzer (Bioclin 100-R729).

### Statistical analysis

The data were initially assessed for homogeneity of variances and normality of the residuals using the Levene and Shapiro–Wilk tests, respectively. The variables that met the assumptions of parametric analysis (glucose, albumin, globulin, and erythrocyte) were subjected to one-way analysis of variance (ANOVA), performed separately according to the comparisons of interest previously established. The effect of CAN concentration was assessed separately at each collection time point (1 h, 24 h, and 24 hR), while the effect of collection time was assessed separately at each CAN concentration (0.00, 0.01, 0.10, and 1.00 mg L⁻¹). This approach was adopted to specifically evaluate the differences between concentrations within each sampling time and between sampling times within each concentration. To compare CAN concentrations within each sampling time, the model $\:{Y}_{ij}=\mu\:+{C}_{i}+{\epsilon\:}_{ij}$, was used, while to compare sampling times within each CAN concentration, the model $\:{Y}_{ij}=\mu\:+{C}_{i}+{\epsilon\:}_{ij}$,was used, where $\:{Y}_{ij}$ represents the observed value of the response variable, $\:\mu\:$ corresponds to the overall mean, $\:{C}_{i}\:\:$represents the effect of CAN concentration, $\:{T}_{i}\:$ represents the effect of sampling time, and $\:{\epsilon\:}_{ij}\:$ corresponds to the random error. When the ANOVA effect was significant, the means were compared using Tukey’s test at the 5% significance level. Variables that did not meet the assumptions of parametric analysis were analyzed using the Kruskal–Wallis test, followed by the Nemenyi test, also at a 5% significance level. All analyses were performed using InfoStat software, version 2014. Detailed statistical results are provided in the Supplementary Material (Tables S1–S4).

## Results

### Cellulose acetate and nanoparticle characterization

**Fig. 1:**
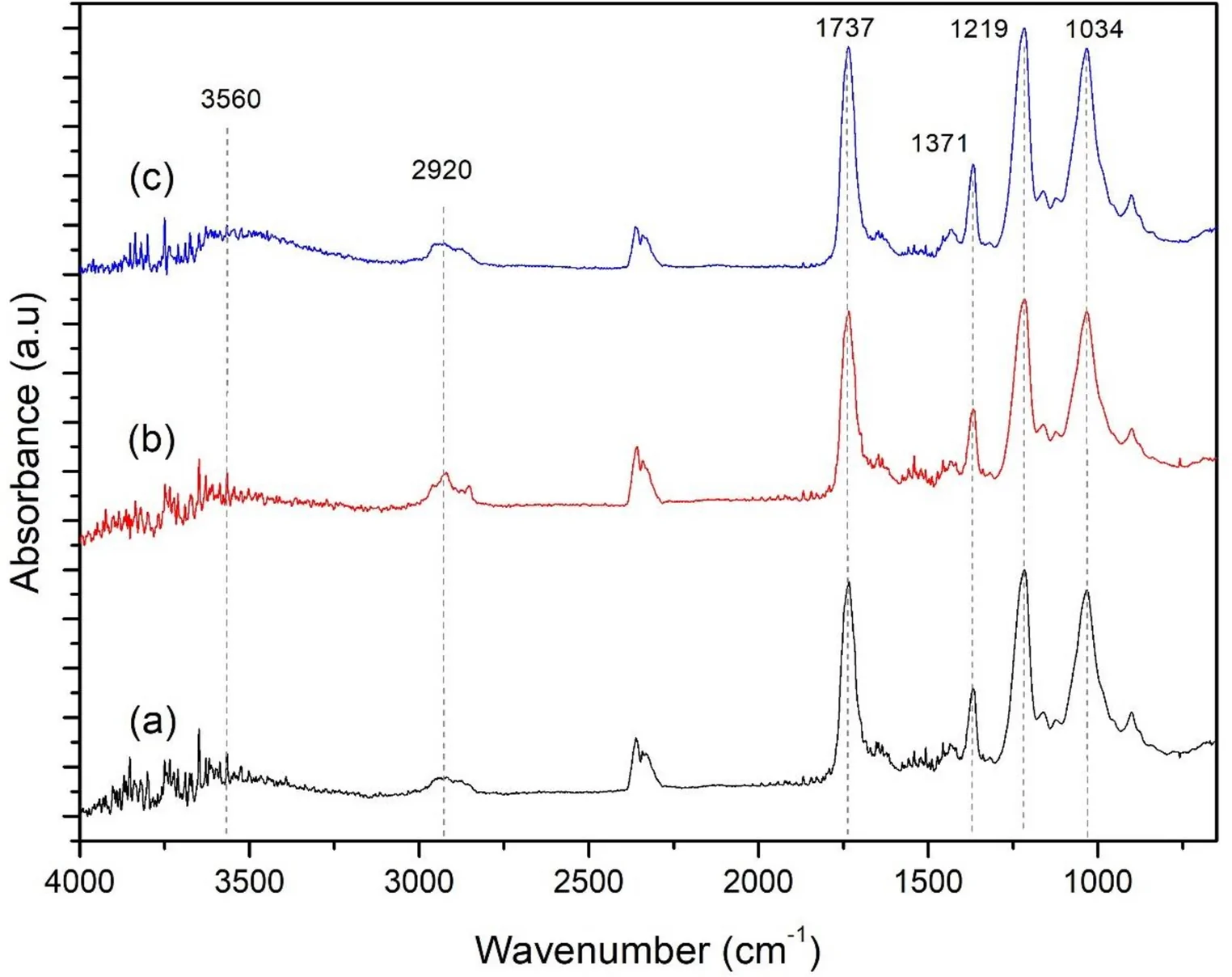
FTIR spectra of cigarette butt filters from three commercial brands: (**a**) Brand 1, (**b**) Brand 2, and (**c**) Brand 3

Figure 1 shows the FTIR spectra obtained from the filters of cigarette butts from three different commercial brands. Broad absorption bands were observed at 3560 cm^-^¹ and 2920 cm^-^¹, corresponding to OH groups and CH stretching vibration, respectively. Absorption peaks centered at 1737, 1371, 1219, and 1034 cm^-^¹, associated with carbonyl stretching, C = O deformation, and C–O–C stretching vibrations, respectively, were also observed. These characteristic bands were consistently present in all samples, reflecting common structural features.

**Fig. 2:**
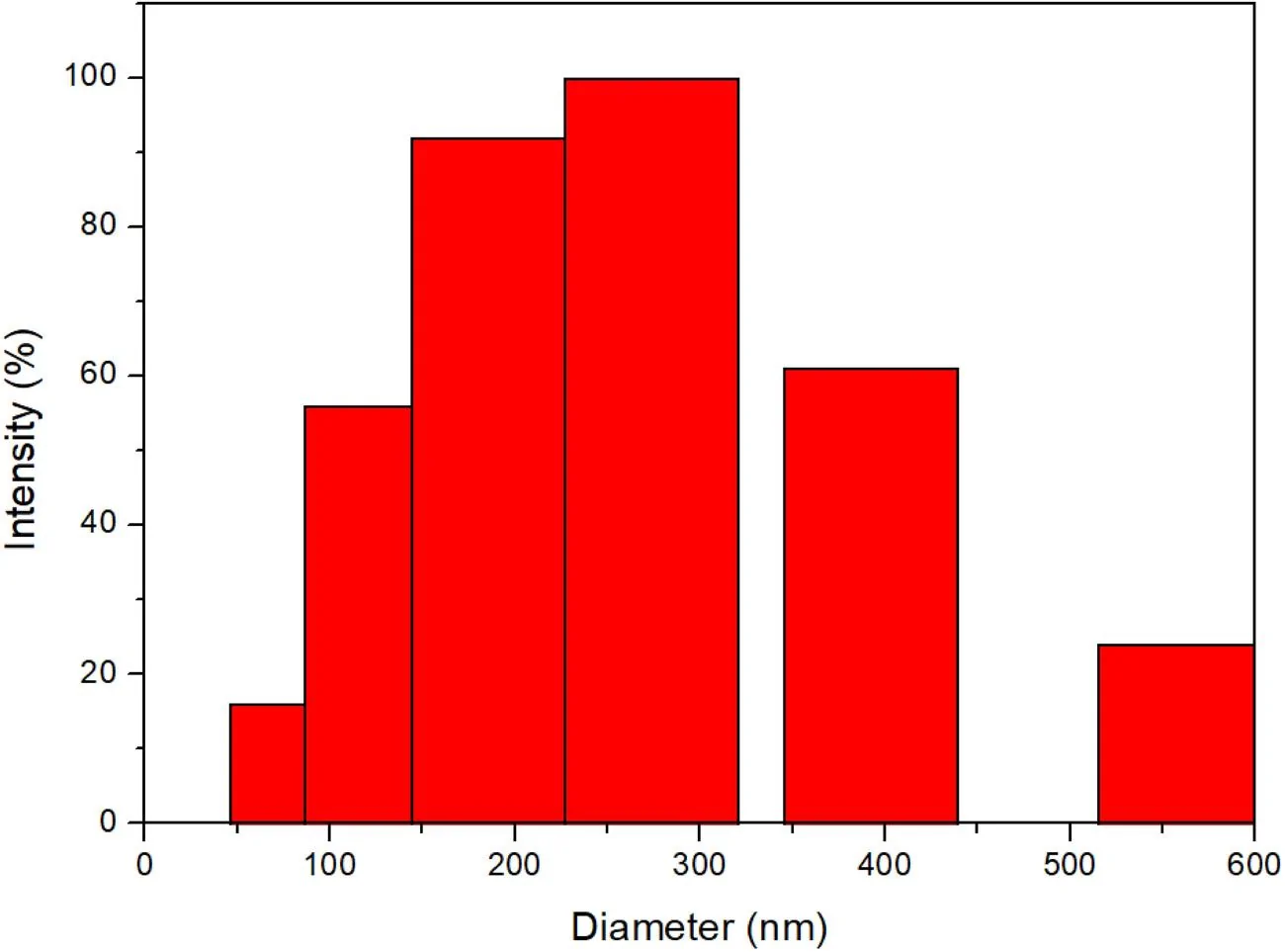
Dynamic Light Scattering (DLS) intensity profile of cellulose acetate nanoparticles (CANs)

**Fig. 3:**
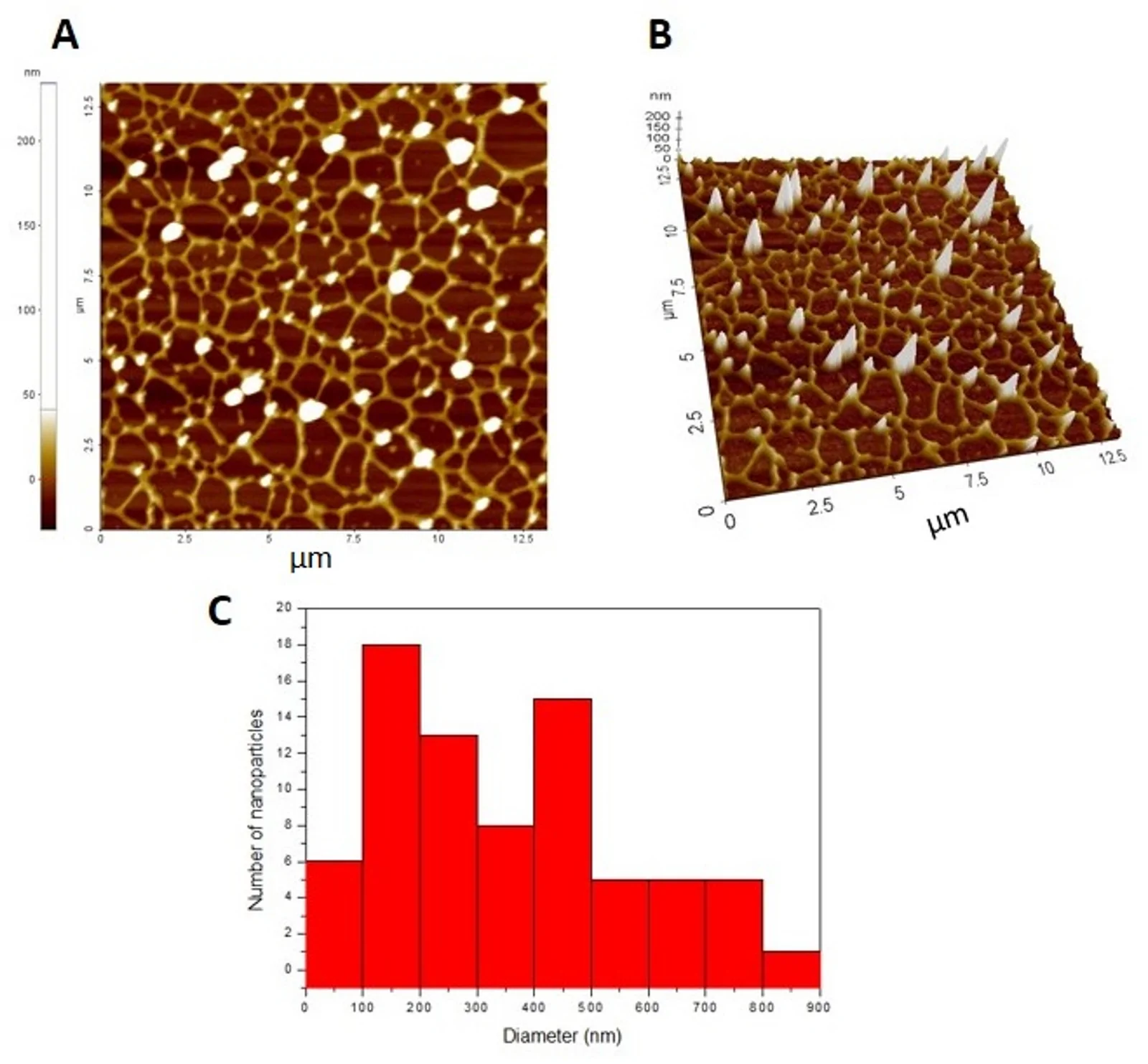
Topographic images (**A** and **B**) of cellulose acetate nanoparticles (CANs) measured by Atomic Force Microscopy (AFM). Particle size histogram (**C**)

The zeta potential of the CANs measured − 21.54 mV, reflecting their surface charge seven days after synthesis (Table 1). The synthesized CANs were characterized by DLS as having an average hydrodynamic diameter of 230.86 ± 3.83 nm and a polydispersity index (PDI) of 0.08 (Table 1). The particle size distribution based on intensity shows diameters ranging from 70 to 600 nm (Fig. 2). ImageJ software processing of the images obtained by AFM (Fig. 3A) calculated the diameters of the observed CANs as ranging from 70 to 880 nm (Fig. 3B). Particle size exhibited a bimodal distribution, with greater numbers of particles between 100 and 200 nm and between 400 and 500 nm and average diameters of 181 ± 13 and 435 ± 24 nm for the first and second distributions, respectively.

**Table 1:** Hydrodynamic diameter and polydispersity index by Dynamic Light Scattering (DLS) and zeta potential (mV) of cellulose acetate nanoparticles (CANs) analyzed seven days after synthesis

Hydrodynamic diameter (nm)	Polydispersity index	Average of zeta potential (mV)
230.7 ± 1.7	0.08	-21.54 mV ± 2.77

Results expressed as mean ± standard deviation of five independent samples, with five measurements performed for each sample (*n* = 5)

### Survival

No mortality was observed during acute-exposure for any of the concentrations of CANs evaluated during the experiment.

### Hematological parameters

**Fig. 4:**
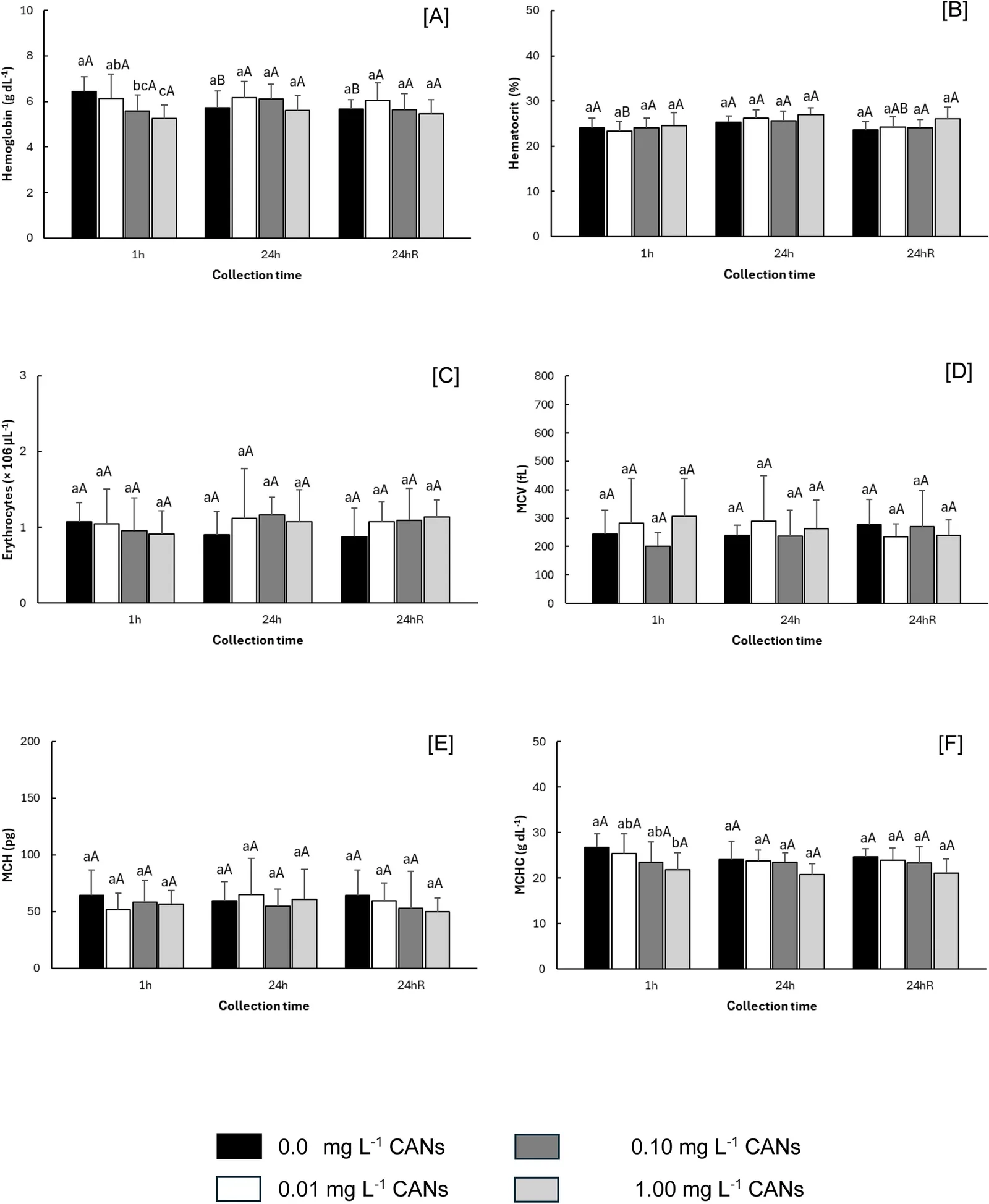
Hematological parameters of *Colossoma macropomum* obtained after exposure to different concentrations of cellulose acetate nanoparticles (CANs) after 1 h, 24 h and 24 h (24 h exposure followed by 24 h in clean water). Results followed by different letters indicate significant difference (*p* < 0.05) by the Kruskal-Wallis test followed by the Nemenyi post hoc test, except for the erythrocytes and mean corpuscular hemoglobin concentrations (MCHC), which were subjected to ANOVA followed by the Tukey’s post hoc test. Lowercase letters indicate a significant difference between treatments at the same sampling time (*p* < 0.05). Capital letters indicate a significant difference for the same treatment in the different sampling times (*p* < 0.05)

After 1 h of exposure, hemoglobin (Fig. 4A) was highest for the control and lowest for 1.00 mg L^-1^ of CANs (*p* < 0.05). After 24 h and 24 h, there were no significant differences among treatments (*p* > 0.05). Comparisons of sampling time for the same treatment found a reduction in hemoglobin after 24 h and 24 h for the control (*p* < 0.05).

No statistically significant differences in hematocrit (Fig. 4B) were observed among concentrations of CANs after 1 h, 24 h and 24 h (*p* > 0.05). However, comparisons of sampling times for the same treatment found decreased hematocrit for 0.01 mg L^-1^ after 1 h (*p* < 0.05).

No statistically significant differences were observed for erythrocytes, MCV and MCH (Figs. 4C, D and E, respectively) (*p* > 0.05). MCHC (Fig. 4F) was reduced, compared to the control (*p* < 0.05), after 1 h of exposure only for the 1.00 mg L^-1^ treatment. No differences were reported among the concentrations of CANs at 24 h and 24 h (*p* > 0.05). Comparisons of sampling times for the same treatment found no significant differences (*p* > 0.05).

### Protein profile

**Fig. 5:**
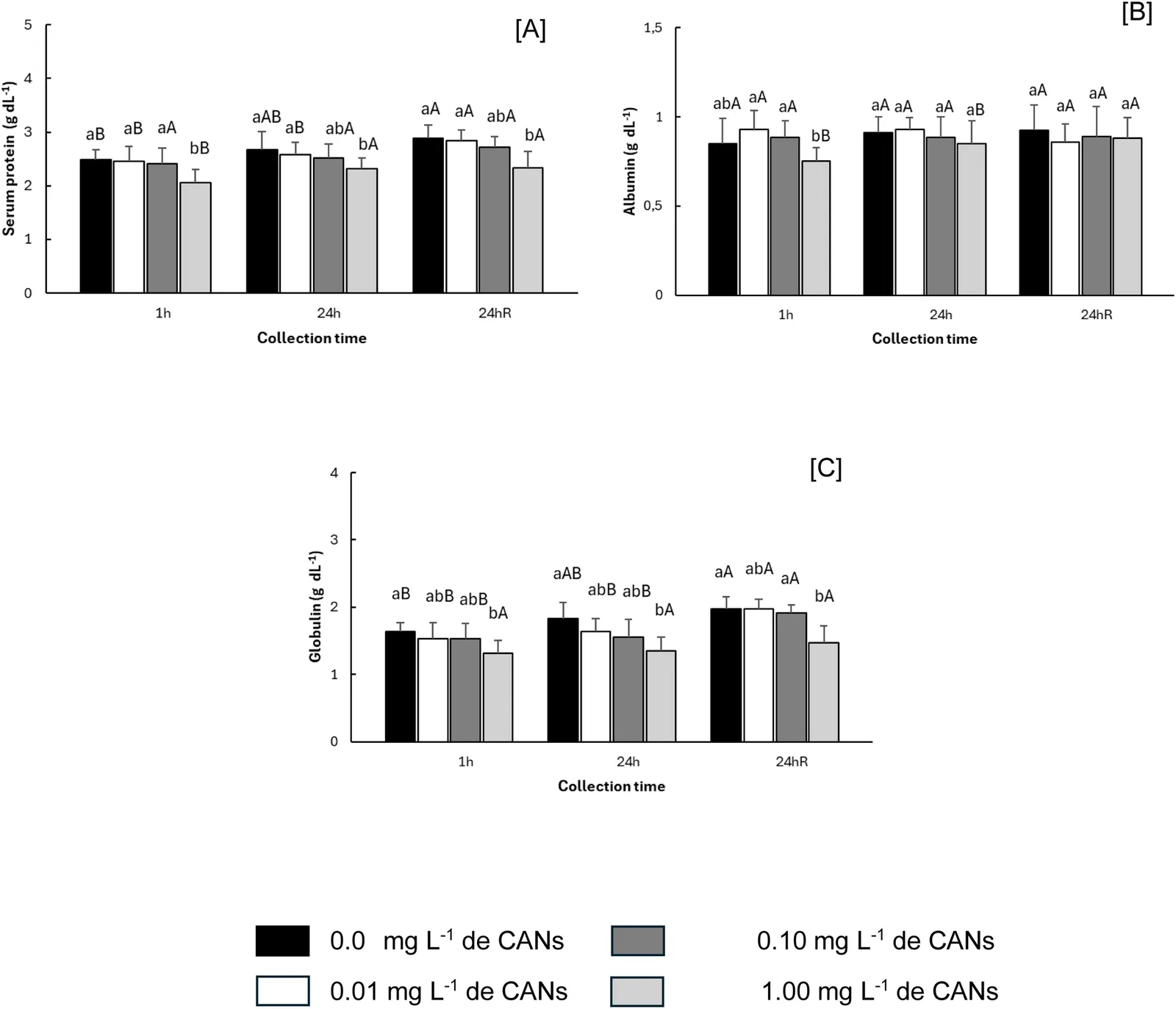
Protein profile of *Colossoma macropomum* obtained after exposure to different concentrations of cellulose acetate nanoparticles (CANs) after 1 h, 24 h and 24 h (24 h exposure followed by 24 h in clean water). Results followed by different letters indicate significant difference (*p* < 0.05) by ANOVA followed by the Tukey’s post hoc test., except for plasma protein, which was subjected to the Kruskal-Wallis test followed by the Nemenyi post hoc test. Lowercase letters indicate a significant difference between treatments in the same sampling time (*p* < 0.05). Capital letters indicate a significant difference for the same treatment in the different sampling times (*p* < 0.05)

After 1 h of exposure, serum protein was reduced for the 1.00 mg L^-1^ of CANs treatment (Fig. 5A) (*p* < 0.05). After 24 h and 24 h, the 1.00 mg L^-1^ treatment had reduced serum protein compared to the control and 0.01 mg L^-1^ (*p* < 0.05). Comparisons of sampling times for the same treatment found the control to have lower serum protein after 1 h compared to 24 h (*p* < 0.05). Treatments 0.01 and 1.00 mg L^-1^ experienced increased serum protein after 24 h. No differences were observed among sampling times for the 0.10 mg L^-1^ treatment (*p* > 0.05).

After 1 h of exposure, albumin (Fig. 5B) was higher for the 0.01 and 0.10 mg L^-1^ treatments compared to 1.00 mg L^-1^ (*p* < 0.05). No statistically significant differences in albumin were observed after 24 h and 24 h (*p* > 0.05). Comparisons of sampling times for the same treatment found an increase for 1.00 mg L^-1^ after 24 h (*p* < 0.05).

Globulin (Fig. 5C) exhibited the same behavior after 1 h, 24 h and 24 h, with lower globulin for 1.00 mg L^-1^ CANs compared to the control (*p* < 0.05). Comparisons of sampling times for the same treatment found treatments 0, 0.01 and 0.10 mg L^-1^ to have higher values only at 24 h (*p* < 0.05); no statistically significant differences were observed among sampling times for the 1.00 mg L^-1^ treatment (*p* > 0.05).

### Biochemical parameters

**Fig. 6:**
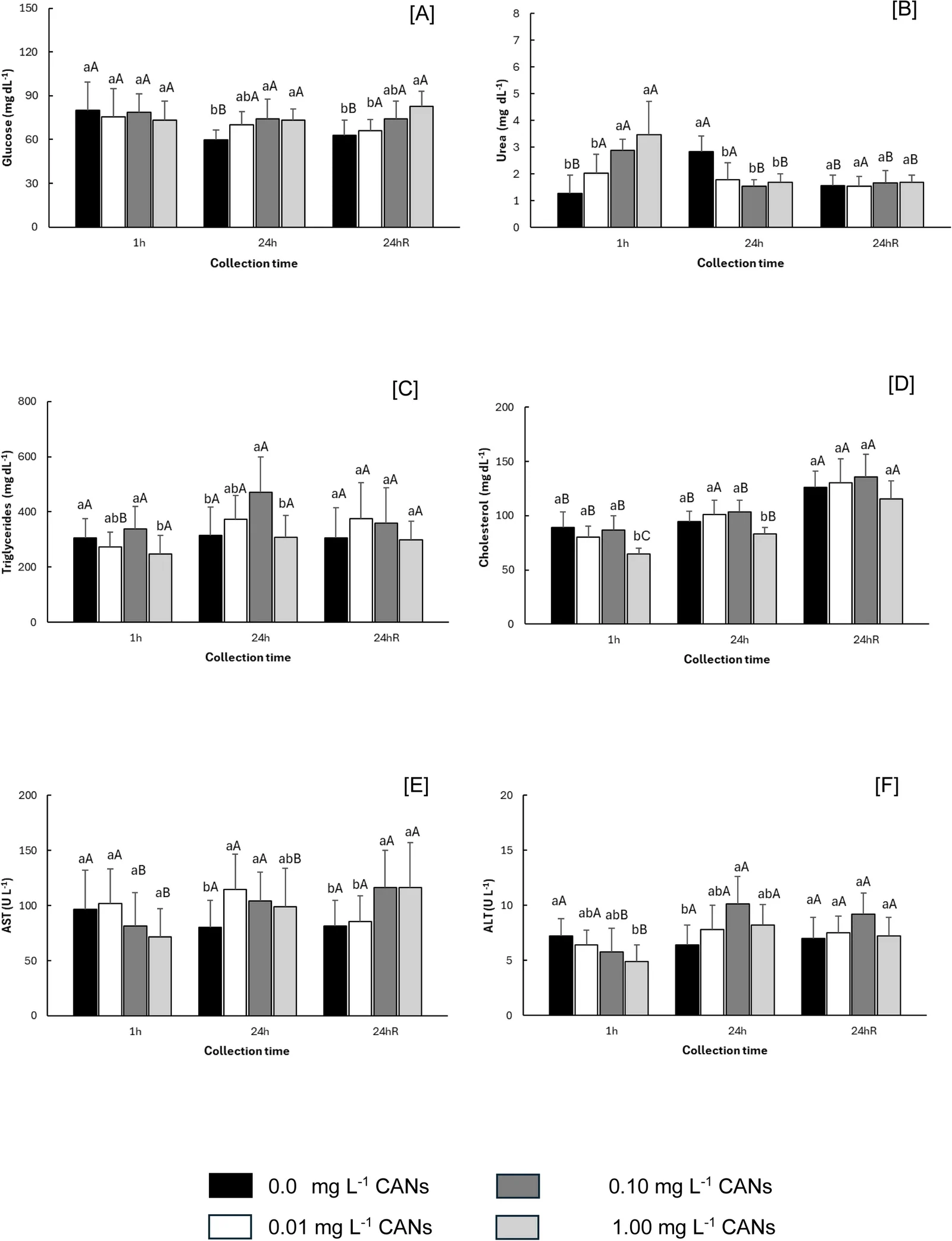
Blood biochemical parameters of *Colossoma macropomum* obtained after exposure to different concentrations of cellulose acetate nanoparticles (CANs) after 1 h, 24 h and 24 h (24 h exposure followed by 24 h in clean water). Results followed by different letters indicate significant difference (*p* < 0.05) by the Kruskal-Wallis test followed by the Nemenyi post hoc test, except for glucose, which was subjected to ANOVA followed by the Tukey’s post hoc test. Lowercase letters indicate a significant difference between treatments at the same sampling time (*p* < 0.05). Capital letters indicate a significant difference for the same treatment in the different sampling times (*p* < 0.05)

Glucose did not differ among treatments (Fig. 6A) after 1 h of exposure (*p* > 0.05). After 24 h, glucose was reduced for the control in relation to the treatments of 0.10 and 1.00 mg L^-1^ of CANs (*p* < 0.05). After 24 h, glucose was lower for the control and 0.01 mg L^-1^ compared to 1.00 mg L^-1^ (*p* < 0.05). Comparisons of sampling times for the control found glucose to be reduced after 24 h and 24 h (*p* < 0.05); the other treatments did not differ statistically among sampling times (*p* > 0.05).

Urea (Fig. 6B) was lower for the control and 0.01 mg L^-1^ treatments after 1 h of exposure (*p* < 0.05). After 24 h, urea was higher for the control than all other treatments (*p* < 0.05). Urea did not differ statistically among treatments after 24 h (*p* > 0.05). Comparisons of sampling times for the same treatment found the control to have higher urea after 24 h (*p* < 0.05). There were no differences in urea among times for the 0.01 mg L^-1^ treatment (*p* > 0.05), while treatments 0.10 and 1.00 mg L^-1^ had increased urea after 1 h (*p* < 0.05).

After 1 h of exposure, triglycerides (Fig. 6C) were lower for the 1.00 mg L-1 of CANs treatment compared to the control and 0.10 mg L-1 (*p* < 0.05). After 24 h, triglycerides were higher for 0.10 mg L^-1^ compared to the control and 1.00 mg L^-1^ (*p* < 0.05). No statistically significant differences in triglyceride levels were observed among treatments after 24 h (*p* > 0.05). Comparisons of sampling times for the same treatment found 0.01 mg L^-1^ to have reduced triglycerides after 1 h (*p* < 0.05).

Cholesterol (Fig. 6D) was reduced for the treatment of 1.00 mg L^-1^ CANs after 1 h and 24 h of exposure; cholesterol did not differ statistically among treatments after 24 h (*p* > 0.05). Comparisons of sampling times for the same treatment revealed higher cholesterol after 24 h for the control and for 0.10 and 1.00 mg L^-1^ (*p* < 0.05).

AST (Fig. 6E) did not differ statistically among treatments after 1 h of exposure (*p* > 0.05), but after 24 h it was reduced for the control compared to 0.01 and 0.10 mg L^-1^ of CANs (*p* < 0.05). After 24 h, AST was reduced for the control and 0.01 mg L^-1^ (*p* < 0.05). Comparisons of sampling times for the same treatment found no differences statistically for the control and 0.01 mg L^-1^ (*p* > 0.05), while lower levels were observed after 1 h for 0.10 mg L^-1^ and higher levels after 24 h for 1.00 mg L^-1^ (*p* < 0.05).

After 1 h of exposure, ALT (Fig. 6F) was lower for the 1.00 mg L^-1^ of CANs treatment compared to the control (*p* < 0.05). After 24 h of exposure, ALT was higher for 0.10 mg L^-1^ compared to the control (*p* < 0.05). ALT did not differ among treatments after 24 h (*p* > 0.05). Comparisons of sampling times for the same treatment found ALT to be reduced after 1 h of exposure for the 0.10 and 1.00 mg L^-1^ treatments (*p* < 0.05).

## Discussion

The FTIR spectra obtained from the cigarette butt filters confirmed the presence of cellulose acetate as the primary constituent. The broad absorption bands observed at 3560 cm^-^¹ and 2920 cm^-^¹ correspond to OH and CH stretching vibrations, which are typical features of the cellulose acetate polymer backbone. The distinct peaks at 1737, 1371, 1219, and 1034 cm^-^¹, associated with carbonyl stretching, C = O deformation, and C–O–C stretching vibrations, further corroborate the presence of ester functionalities characteristic of cellulose acetate. All absorption bands obtained for cigarette butt samples are characteristic of commercial cellulose acetate already reported in other studies (Teixeira et al. 2023; Gonçalves et al. 2019). The similarity of the FTIR spectra for all analyzed samples, regardless of the cigarette brand, indicates a consistent composition in commercial cigarette filters. These findings are consistent with the study by Wilkinson et al. (2025), who characterized smoked and unsmoked cigarette butts by FTIR and compared their spectra with those of commercial cellulose acetate. In their work, the characteristic absorption bands of cellulose acetate were observed at 1734, 1367, 1215, and 1031 cm^-^¹, confirming the presence of CA in all samples.

The − 21.54 mV zeta potential of the nanoparticles indicates moderate stability. Zeta potential values lower than − 30 mV and higher than + 30 mV suggest strong electrostatic repulsion between particles, which prevents the formation of agglomerates (Bhattacharjee et al. 2016). The DLS intensity distribution showed particle diameters ranging from approximately 70 to 600 nm, with the highest intensity observed for particles between approximately 150 and 300 nm. This range encompasses the mean hydrodynamic diameter of 230.7 ± 1.7 nm obtained directly from the DLS instrument software. The DLS analysis also revealed a PDI of 0.08. PDI values higher than 0.4 indicate a highly polydisperse particle size distribution (Bhattacharjee 2016). This result was confirmed by the particle size dispersion observed both in the DLS intensity distribution graph and in the histogram obtained by AFM. The histogram obtained through AFM image analysis shows a bimodal distribution, with the first distribution being indicative of the formation of individual particles with an average diameter of 181 ± 13 nm. The average particle size obtained by AFM analysis for this distribution was smaller than those obtained by DLS (230 and 262 nm). This difference is expected since DLS measures hydrodynamic diameter while AFM measures dry particle diameter, and it is common for AFM to obtain smaller values than those reported by DLS. The second distribution of the histogram, with an average diameter of 435 ± 24 nm, suggests the formation of particle agglomerates. Lesaine et al. (2021) demonstrated that colloidal suspensions subjected to slow drying processes present significant aggregate formation due to longer residence time in the concentrated liquid state. Thus, the agglomerates could have been obtained from the drying process at room temperature for AFM analysis. The synthesized nanoparticles exhibited sizes compatible with the proposed applications in biological and agricultural systems, such as vehicles for the release of drugs and agricultural inputs (Yusuf et al. 2023). In addition, the sizes obtained are within the range capable of achieving cellular penetration, thereby causing damage to the gills and crossing the blood-brain barrier of juvenile fish (Guerrera et al. 2021). To ensure the size of the nanoparticles without agglomeration, the experiments were carried out between five and seven days after preparation.

It is important to note that exposure concentrations were based on nominal values, as analytical verification of CAN levels in the experimental water was not technically feasible due to the high content of dissolved organic matter and suspended particles in the culture system, which interfere with nanoparticle detection and physicochemical analyses. The zeta potential (− 21.54 mV), determined under controlled conditions for initial characterization, indicates moderate colloidal stability; therefore, under experimental exposure conditions, some degree of aggregation and/or sedimentation may have occurred. Such behavior is consistent with nanoparticle dynamics in natural aquatic environments, where ionic strength and organic matter significantly influence colloidal stability (Li et al. 2022). Consequently, potential changes in dispersion over time should be considered an factor when interpreting biological responses. Moreover, the analytical challenges associated with the detection and physicochemical characterization of nanoscale plastic particles in complex environmental matrices are well recognized in the literature, as the presence of dissolved organic matter, ions, and suspended particles significantly interferes with separation, identification, and reliable quantification of nanoplastics in real samples (Zhang et al.,^2022^).

Exposure of fish to pollutants, such as plastic nanoparticles, can compromise their homeostasis, triggering changes in blood parameters (Sayed and Soliman 2018; Guerrera et al. 2021). Hematological and biochemical parameters are essential indicators that reflect the health condition of fish (Thummabancha et al. 2016). The present study found a decrease in hemoglobin and MCHC 1 h after exposure to the highest concentration of CANs, while no statistically significant differences were observed in these parameters after 24 h. However, no statistically significant differences were observed in hematocrit, erythrocytes, MCV and MCH for any of the evaluated periods. Vineetha et al. (2024) observed reduced hemoglobin levels for juvenile *Oreochromis niloticus* exposed to polystyrene nanoparticles at the concentrations of 0.10, 1.00, and 10 mg L^-^¹. Similar results were observed by Lee et al. (2023) with decreases in these parameters for *Pseudobagrus fulvidraco* when exposed to high concentrations of high-density polyethylene microplastics. Hamed et al. (2019) also found reductions in hemoglobin and MCHC when the same species was exposed to microplastic concentrations from 1 to 100 mg L^-^¹.

Specific data on environmental concentrations of CANs in aquatic environments are still limited, reflecting the methodological difficulty in quantifying particles at the nanoscale. In general, environmentally realistic concentrations for plastic microparticles range from approximately 0.001 mg L⁻¹ to 0.023 mg L⁻¹ (Sussarellu et al. 2016; Lenz et al. 2016). For nanoparticles, estimates indicate values of up to 0.015 mg L⁻¹ as high environmental concentrations (Al-Sid-Cheikh et al. 2018). This wide variation highlights the uncertainty that still exists regarding the actual concentrations of nanoplastics in the environment. In this context, the concentrations used in the present study were selected to represent an experimental gradient composed of an estimated environmental scenario (0.01 mg L⁻¹), a possible future scenario of intensified contamination (0.10 mg L⁻¹), and an extreme scenario (1.00 mg L⁻¹), allowing the evaluation of physiological responses under different levels of exposure. In addition, similar concentration ranges have already been used in experimental studies (Brandts et al. 2021; Hayati et al. 2022; Marana et al. 2022; Mohammadzadeh et al. 2024).

The present study found that serum protein and globulin levels decreased after 1 h of exposure to 1.00 mg L^-^¹ of CANs, and that this decrease persisted after 24 h of exposure. Serum proteins play a crucial role in maintaining osmotic balance in the blood by preventing excessive fluid outflow from the circulation (Girling et al. 2015; Espinosa et al. 2017). In response to stress, adrenaline is released into the circulation, which can promote increased gill permeability and cause changes in blood plasma volume (Mazeaud et al. 1977). It is possible that continuous exposure to CANs altered protein metabolism, resulting in reduced serum proteins and/or increased utilization of these proteins. In addition, the reduction in globulins may be related to changes in the immune response described in the literature (Atef et al. 1991; Nayak et al. 2004; Karami et al., 2016), although the present study did not evaluate specific immunological biomarkers that would allow confirmation of this mechanism.

Initial exposure to CANs did not result in an immediate glycemic response in the present study. However, after 24 h, juvenile *C. macropomum* exposed to 0.10 and 1.00 mg L^-^¹ of CANs had elevated glucose levels, suggesting that the exposure to CANs altered glycemic metabolism. Even after transfer to clean water, juveniles exposed to the higher concentration were unable to reduce these elevated levels, which may reflect a prolonged stress response. Glucose is widely recognized as an indirect stress biomarker in fish (Abbaszadeh et al. 2024). Plasma glucose homeostasis is controlled by the balance between its synthesis and its storage in the form of glycogen.(Sharab 2015) Exposure of fish to toxic substances can lead to increased plasma glucose levels, since these organisms mobilize their energy reserves through the release of catecholamines and corticosteroids, stimulating glycogenolysis to meet energy demand (Barton and Iwama 1991). Furthermore, such exposure can reduce glucose uptake and activate gluconeogenesis (Hamed et al. 2019; Kim et al. 2021).

Exposure of juvenile *C. macropomum* to 0.10 and 1.00 mg L^-^¹ of CANs impacted urea levels in the body within the first hour. Urea is a nitrogenous byproduct resulting from protein metabolism (Salazar 2014), originating from both the degradation of dietary proteins and the breakdown of tissues (Schutz^ 2011^). Under stress conditions, proteolysis can be intensified (Herrera et al. 2019), as proteins play an essential role in maintaining cellular homeostasis and act as an alternative energy source (Amirkolaie 2011). A reduction in urea levels was observed for all concentrations of CANs after 24 h of exposure. It is possible that these variations are related to adjustments in nitrogen metabolism described in the literature (Ip and Chew 2010). This effect is reinforced by the fact that blood urea levels returned to normal after hours in clean water (24 h). Thus, the changes observed in urea levels were transient under the experimental conditions evaluated.

The present study found that exposure to 1.00 mg L^-^¹ of CANs caused a reduction in circulating triglyceride and cholesterol levels in juveniles 1 h after exposure. A similar effect was observed by Liao et al. (2025) with a reduction in cholesterol and triglycerides when juvenile *O. niloticus* were exposed to polystyrene nanoparticles. Exposure to plastics can compromise fish cell membranes, alter transmembrane gradients, and induce or inhibit the activity of enzymes involved in lipid metabolism, resulting in variations in circulating triglyceride and cholesterol levels (Banaee et al. 2019; Kim et al. 2021). However, an increase in triglyceride levels was found after 24 h for juveniles of exposed to 0.10 mg L^-^¹. According to Kim et al. (2021), an increase in plasma triglyceride levels may be a physiological response of organisms to meet energy demand and mitigate the toxic effects of these pollutants. When juveniles remained in clean water, triglyceride and cholesterol levels recovered.

The present study found a reduction in ALT activity after 1 h of exposure to 1.00 mg L⁻¹ of CANs. After 24 h, an increase in AST was observed in the groups exposed to 0.01 and 0.10 mg L⁻¹, and an increase in ALT in the group exposed to 0.10 mg L⁻¹. In contrast to ALT, which normalized after the recovery period, AST remained elevated at higher concentrations, indicating that the enzymatic response was not completely reversed in the evaluated interval. Changes in AST and ALT levels have already been reported in several species, such as *O. niloticus*, *Danio rerio*, *Larimichthys crocea*, and *Pseudobagrus fulvidraco*, when exposed to micro- and nanoplastics (Hamed et al. 2019; Umamaheswari et al. 2021; Lai et al. 2021; Lee et al. 2023). When fish are exposed to environmental contaminants, there may be an increase in the release of AST and ALT enzymes into the bloodstream, possibly due to increased permeability of hepatocyte membranes (Lee et al. 2023). These enzymes also play an important role in the activation of gluconeogenesis (Bonifacio et al. 2017; Shivashri et al. 2013; Lee et al. 2023). However, in this study, the absence of a consistent dose-response pattern and the predominantly transient nature of the changes suggest that exposure to CANs was associated with mild and partially reversible physiological disturbances under the experimental conditions evaluated. Furthermore, the observed fluctuations may reflect metabolic adjustments in response to exposure to nanoparticles, indicating an adaptive physiological response.

This study used PVA as a surfactant during the synthesis of CANs. Although PVA was not intentionally added as an exposure compound, this polymer may have remained in the final suspension used in the treatments. However, it is important to note that a 2% concentration of PVA was used during the synthesis step and does not necessarily correspond to the concentration to which the juveniles were exposed. No quantification of residual PVA in the final CAN suspension was performed, so it was not possible to experimentally determine the concentration of PVA to which the juveniles were actually exposed. Based on the composition used in the synthesis (1,000 mg of CA in 100 mL of 2% PVA solution), the theoretical maximum PVA ratio was 2:1. Thus, assuming 100% retention of PVA, the theoretical maximum PVA concentrations would be 0.02, 0.20, and 2.00 mg L⁻¹ in the treatments containing 0.01, 0.10, and 1.00 mg L⁻¹ of CANs, respectively. Nigro et al. (2022) observed no toxic effects of PVA on *Daphnia magna* and *Danio rerio* embryos at concentrations up to 1 mg L⁻¹. Zicarelli et al. (2024) evaluated different types of PVA at concentrations ranging from 0.1 to 100 mg L⁻¹ in D. rerio embryos. Although they did not observe significant effects on mortality or the occurrence of malformations, significant changes in heart rate were observed for the PVA with the lowest molecular weight at concentrations of PVA ≥ 5 mg L⁻¹. Zhang et al. (2024) observed adverse effects in D. rerio exposed to 5 mg L⁻¹ of PVA, including alterations in development and in the hypothalamic–pituitary–thyroid axis. However, this concentration is higher than the theoretical maximum PVA concentration (2.00 mg L⁻¹) estimated for the present study. Given that the theoretical maximum concentration of PVA in this study could reach 2.00 mg L⁻¹, it is not possible to completely rule out a potential contribution of PVA to the observed physiological responses, especially in the treatment group with the highest concentration of CANs. This limitation should be taken into account when interpreting the results, since a control group containing PVA at a concentration corresponding to that of the vehicle was not included. Future studies should consider including control groups with concentrations of surfactant similar to those used in the synthesis of nanoparticles, thereby allowing for a more accurate assessment of the surfactant’s contribution to the observed effects.

In general, the physiological responses observed in this study did not show a clear progressive relationship between increasing concentration and effect intensity. Some changes were predominantly transient under the conditions evaluated. Furthermore, in certain cases, the intermediate concentration (0.10 mg L⁻¹) triggered responses similar to or even more pronounced than those observed at the highest concentration (1.00 mg L⁻¹). Similar patterns have been described in ecotoxicological studies involving plastic contaminants, in which higher levels of exposure do not necessarily result in more intense effects (Parker et al. 2021). Similarly, Oliveira et al. (2025) also did not observe a proportional relationship between the increase in polypropylene microparticle concentrations and the physiological responses of blood parameters in juvenile *Piaractus brachypomus*. In the present study, the absence of mortality and the recovery of several parameters after the clearance period suggest that the changes observed reflect acute and potentially reversible physiological adjustments. In addition, hematological and biochemical parameters in fish exhibit intrinsic biological variation (Ahmed et al. 2020), which may influence the magnitude of the responses detected and contribute to specific differences among the evaluated concentrations.

However, prolonged exposure to nanoplastics may lead to a gradual increase in their concentration in fish tissues, with subsequent distribution to different organs, such as the liver, which plays an important role in the metabolism and processing of potentially toxic substances (Thakur et al. 2025). During prolonged exposure, for example, the continuous presence of nanoparticles may lead to cellular stress and physiological changes that are undetectable in the early stages of exposure (Yan et al. 2026). Therefore, the responses observed after 1 h and 24 h should be interpreted in the context of acute-exposure to CANs and cannot necessarily be extrapolated to longer exposure periods. Additional studies involving prolonged exposure to CANs are needed to determine how their effects may change over time.

The findings of this study broaden our understanding of the acute physiological effects of CANs in juvenile *C. macropomum* and lay the foundation for further research. Future studies may integrate tissue-level analyses and mechanistic biomarkers, such as oxidative stress parameters and histopathological assessments, to further elucidate the cellular processes involved. It is important to consider that the absence of statistically significant differences for some parameters should be interpreted in light of the experimental conditions and the number of experimental replicates evaluated in this study.In addition, increasing the number of individuals and applying different experimental approaches may expand the analytical robustness and ecological extrapolation of the results, contributing to the advancement of ecotoxicological assessments involving nanoplastics.

## Conclusions

The study demonstrated that the exposure of juvenile tambaqui (*Colossoma macropomum*) to cellulose acetate nanoparticles affected several physiological parameters. Although exposure caused temporary changes in blood biochemistry and hematology, partial recovery was observed after 24 h in clean water. These results indicate that cellulose acetate may induce acute and partially reversible physiological modulations in the short term. However, further studies are needed to elucidate the possible effects of prolonged exposure to these nanoparticles.

## Supplementary information

Below is the link to the electronic supplementary material.

Supplementary Material 1

Supplementary Material 2

Supplementary Material 3

Supplementary Material 4

## Data Availability

No datasets were generated or analysed during the current study.
