# Supplementary material for: Cellulose acetate nanoparticles: impacts of concentration and exposure time on the biochemistry and hematology of juvenile Colossoma macropomum
Source: Ecotoxicology. 2026 Oct 23;35(9):189. doi: 10.1007/s10646-026-03174-w (PMC13694297; doi:10.1007/s10646-026-03174-w)
Supplement: Supplementary file 1 [file 10646_2026_3174_MOESM1_ESM.docx]

| **Variable** | **Comparison** | **Condition** | **df** | **F** | **H** | **ε^2^** | **η^2^** | **p-value** |
| --- | --- | --- | --- | --- | --- | --- | --- | --- |
| **Glucose** | Among sampling times | 0.00 | 2.26 | 6.18 | - | - | 0.322 | 0.0064 |
|  |  | 0.01 | 2.26 | 1.28 | - | - | 0.089 | 0.2958 |
|  |  | 0.10 | 2.27 | 0.44 | - | - | 0.031 | 0.6497 |
|  |  | 1.00 | 2.27 | 2.68 | - | - | 0.166 | 0.0866 |
|  | Among CANs concentrations | 1h | 3.36 | 0.32 | - | - | 0.026 | 0.8133 |
|  |  | 24h | 3.35 | 4.22 | - | - | 0.266 | 0.0120 |
|  |  | 24hR | 3.35 | 7.12 | - | - | 0.379 | 0.0007 |
| **Urea** | Among sampling times | 0.00 | 2 | - | 14.89 | 0.620 | - | 0.0006 |
|  |  | 0.01 | 2 | - | 2.15 | 0.074 | - | 0.3413 |
|  |  | 0.10 | 2 | - | 18.64 | 0.666 | - | 0.0001 |
|  |  | 1.00 | 2 | - | 14.58 | 0.561 | - | 0.0007 |
|  | Among CANs concentrations | 1h | 3 | - | 19.70 | 0.563 | - | 0.0002 |
|  |  | 24h | 3 | - | 17.24 | 0.466 | - | 0.0006 |
|  |  | 24hR | 3 | - | 1.28 | 0.36 | - | 0.7300 |
| **Triglycerides** | Among sampling times | 0.00 | 3 | - | 0.62 | 0.021 |  | 0.7327 |
|  |  | 0.01 | 2 | - | 6.55 | 0.015 | - | 0.0378 |
|  |  | 0.10 | 2 | - | 5.71 | 0.220 | - | 0.0574 |
|  |  | 1.00 | 2 | - | 5.33 | 0.184 | - | 0.0697 |
|  | Among CANs concentrations | 1h | 3 | - | 8.63 | 0.221 | - | 0.0346 |
|  |  | 24h | 3 | - | 11.41 | 0.308 | - | 0.0097 |
|  |  | 24hR | 3 | - | 2.80 | 0.076 | - | 0.4243 |
| **Cholesterol** | Among sampling times | 0.00 | 2 | - | 17.53 | 0.604 | - | 0.0002 |
|  |  | 0.01 | 2 | - | 17.74 | 0.634 | - | 0.0001 |
|  |  | 0.10 | 2 | - | 18.52 | 0.639 | - | 0.0001 |
|  |  | 1.00 | 2 | - | 25.,30 | 0.872 | - | <0.0001 |
|  | Among CANs concentrations | 1h | 3 | - | 19.86 | 0.509 | - | 0.0002 |
|  |  | 24h | 3 | - | 16.76 | 0.441 | - | 0.0008 |
|  |  | 24hR | 3 | - | 5.87 | 0.151 | - | 0.1180 |
| **AST** | Among sampling times | 0.00 | 2 | - | 1.95 | 0.067 | - | 0.3762 |
|  |  | 0.01 | 2 | - | 3.74 | 0.129 | - | 0.1539 |
|  |  | 0.10 | 2 | - | 7.62 | 0.272 | - | 0.0220 |
|  |  | 1.00 | 2 | - | 8.86 | 0.306 | - | 0.0119 |
|  | Among CANs concentrations | 1h | 3 | - | 6.50 | 0.171 | - | 0.0895 |
|  |  | 24h | 3 | - | 8.65 | 0.222 | - | 0.0342 |
|  |  | 24hR | 3 | - | 10.82 | 0.277 | - | 0.0126 |
| **ALT** | Among sampling times | 0.00 | 2 | - | 1.66 | 0.057 | - | 0.4191 |
|  |  | 0.01 | 2 | - | 2.44 | 0.084 | - | 0.2745 |
|  |  | 0.10 | 2 | - | 12.43 | 0.460 | - | 0.0018 |
|  |  | 1.00 | 2 | - | 12.33 | 0.457 | - | 0.0018 |
|  | Among CANs concentrations | 1h | 3 | - | 8.23 | 0.211 | - | 0.0347 |
|  |  | 24h | 3 | - | 10.62 | 0.287 | - | 0.0127 |
|  |  | 24hR | 3 | - | 7.,01 | 0.189 | - | 0.0632 |

**Table S2.** Summary of statistical analyses performed for blood biochemical parameters. Results are presented as the test statistic (F for ANOVA or H for Kruskal–Wallis), degrees of freedom (df), exact p-values, and effect sizes (η² for ANOVA and ε² for Kruskal–Wallis).

Tukey's test was used for multiple comparisons following significant ANOVA results, whereas Nemenyi's test was used following significant Kruskal-Wallis results. A dash (-) indicates that the statistic was not applicable.
