# Supplementary material for: Cellulose acetate nanoparticles: impacts of concentration and exposure time on the biochemistry and hematology of juvenile Colossoma macropomum
Source: Ecotoxicology. 2026 Oct 23;35(9):189. doi: 10.1007/s10646-026-03174-w (PMC13694297; doi:10.1007/s10646-026-03174-w)
Supplement: Supplementary file 2 [file 10646_2026_3174_MOESM2_ESM.docx]

|  |  |  |  |  | **IC95%** |  |  |  |  |  |  |  |
| --- | --- | --- | --- | --- | --- | --- | --- | --- | --- | --- | --- | --- |
| **Variable** | **0 mg L^-1^**  **1 h** | **0 mg L^-1^ ^–^**  **24 h** | **0 mg L^-1^**  **24 hR** | **0,01 mg L^-1^**  **1 h** | **0,01 mg L^-1^ 24 h** | **0,01 mg L^-1^ 24 hR** | **0,10 mg L^-1^**  **1 h** | **0,10 mg L^-1^**  **24 h** | **0,10 mg L^-1^**  **24 hR** | **1,00 mg L^-1^**  **1 h** | **1,00 mg L^-1^**  **24 h** | **1,00 mg L^-1^**  **24 hR** |
| **Hemoglobin (g dL^-1^)** | 5,95–6,90 | 5,21–6,72 | 5,39–5,97 | 5,38–6,89 | 5,67–6,69 | 5,50–6,60 | 5,09–6,09 | 5,66–6,58 | 3,90–6,36 | 4,83–5,67 | 5,17–6,07 | 5,04–5,91 |
| **Glucose (mg dL^-1^)** | 65,90–93,93 | 54,46–65,01 | 55,69–70,45 | 61,83–89,46 | 63,26–76,52 | 45,88–74,35 | 69,72–87,64 | 64,45–83,72 | 65,13–82,85 | 64,18–82,73 | 67,61–78,53 | 75,30–90,15 |
| **Triglycerides (mg dL^-1^)** | 255,51–354,70 | 240,99–468,98 | 228,14–384,69 | 233,86–311,37 | 312,71–434,49 | 281,24–469,19 | 278,55–396,02 | 384,22–646,46 | 202,93–547,99 | 198,07–295,93 | 252,26–365,51 | 248,19–346,67 |
| **Cholesterol (mg dL^-1^)** | 78,95–99,26 | 87,43–101,42 | 115,56–136,82 | 73,34–87,61 | 90,93–111,36 | 114,05–146,18 | 77,11–96,28 | 95,62–111,07 | 120,97–150,96 | 61,07–68,70 | 78,75–87,29 | 103,27–127,28 |
| **Hematocrit (%)** | 22,61–25,59 | 24,32–26,35 | 22,11–25,00 | 21,79–24,81 | 24,80–27,65 | 22,55–25,85 | 20,73–25,67 | 23,88–27,24 | 22,68–26,72 | 22,44–26,56 | 25,99–28,01 | 24,33–27,87 |
| **Serum protein (g dL^-1^)** | 2,31–2,67 | 2,56–2,92 | 1,84–6,30 | 2,26–2,66 | 2,40–2,73 | 2,28–2,97 | 2,18–2,64 | 2,18–2,69 | 2,01–3,02 | 1,87–2,25 | 1,96–2,44 | 2,11–2,57 |
| **Albumin (g dL^-1^)** | 0,72–0,98 | 0,85–0,98 | 0,83–1,03 | 0,85–1,01 | 0,63–1,05 | 0,79–0,93 | 0,82–0,95 | 0,80–0,97 | 0,76–1,02 | 0,69–0,81 | 0,76–0,94 | 0,80–0,96 |
| **Globulin(g dL^-1^)** | 1,50–1,77 | 1,65–2,00 | 0,89–5,40 | 1,35–1,71 | 1,49–1,78 | 1,44–2,10 | 1,34–1,71 | 1,36–1,75 | 1,77–2,05 | 1,17–1,46 | 1,19–1,51 | 1,28–1,66 |
| **Urea (mg dL^-1^)** | 0,30–3,71 | 2,47–4,21 | 1,22–2,34 | 1,53–2,55 | 1,32–2,25 | 1,27–1,82 | 2,59–3,19 | 1,38–1,72 | 1,31–2,03 | 3,07–6,03 | 1,49–1,91 | 1,52–1,88 |
| **ALT (U L^-1^)** | 6,04–8,36 | 5,09–7,71 | 5,65–8,35 | 5,43–7,37 | 6,23–9,37 | 6,42–8,58 | 4,30–7,30 | 8,21–14,19 | 7,22–13,98 | 3,81–5,99 | 6,75–11,45 | 5,98–9,82 |
| **AST (U L^-1^)** | 70,99–122,01 | 63,33–97,67 | 64,60–98,20 | 79,72–124,28 | 91,35–137,65 | 68,98–102,22 | 60,12–125,48 | 84,82–122,98 | 92,01–140,39 | 53,48–89,72 | 73,73–124,07 | 86,73–145,67 |
| **Erythrocytes (× 106 μL^-1^)** | 0,89–1,25 | 0,68–1,12 | 0,60–1,15 | 0,69–1,40 | 0,64–1,59 | 0,88–1,26 | 0,63–1,29 | 1,00–1,33 | 0,76–1,42 | 0,69–1,13 | 0,77–1,37 | 0,98–1,30 |
| **MCV (fL)** | 183,00–303,19 | 205,96–273,32 | 202,50–351,42 | 159,77–403,14 | 157,71–422,32 | 203,17–266,71 | 155,74–244,33 | 168,27–307,17 | 172,21–367,04 | 209,98–401,84 | 184,80–340,74 | 198,66–278,65 |
| **MCH (pg)** | 48,81–80,51 | 45,30–73,43 | 47,05–81,71 | 37,69–65,30 | 40,12–89,54 | 48,25–70,95 | 41,95–74,28 | 44,43–65,38 | 28,02–78,03 | 47,32–65,66 | 42,22–79,63 | 41,70–58,43 |
| **MCHC (g dL^-1^)** | 24,68–28,87 | 20,95–27,17 | 23,26–25,98 | 22,11–28,75 | 21,85–25,59 | 21,85–25,93 | 20,09–26,90 | 21,85–25,02 | 20,44–26,11 | 19,00–24,50 | 19,17–22,51 | 18,96–23,28 |

**Table S1.** 95% confidence intervals (95% CI) of blood variables for each combination of CAN concentration and collection time.
