# Supplementary material for: Cellulose acetate nanoparticles: impacts of concentration and exposure time on the biochemistry and hematology of juvenile Colossoma macropomum
Source: Ecotoxicology. 2026 Oct 23;35(9):189. doi: 10.1007/s10646-026-03174-w (PMC13694297; doi:10.1007/s10646-026-03174-w)
Supplement: Supplementary file 3 [file 10646_2026_3174_MOESM3_ESM.docx]

| **Variable** | **Comparison** | **Condition** | **df** | **F** | **H** | **ε^2^** | **η^2^** | **p-value** |
| --- | --- | --- | --- | --- | --- | --- | --- | --- |
| **Serum protein** | Among sampling times | 0.00 | 2 | - | 7.12 | 0.310 | - | 0.0283 |
|  |  | 0.01 | 2 | - | 8.93 | 0.343 | - | 0.0115 |
|  |  | 0.10 | 2 | - | 4.95 | 0.206 | - | 0.0841 |
|  |  | 1.00 | 2 | - | 7.29 | 0.292 | - | 0.0260 |
|  | Among CAN concentrations | 1h | 3 | - | 9.36 | 0.284 | - | 0.0247 |
|  |  | 24h | 3 | - | 12.84 | 0.367 | - | 0.0050 |
|  |  | 24hR | 3 | - | 13.64 | 0.440 | - | 0.0034 |
| **Albumin** | Among sampling times | 0.00 | 2.24 | 0.88 | - | - | 0.075 | 0.4296 |
|  |  | 0.01 | 2.26 | 1.84 | - | - | 0.115 | 0.1786 |
|  |  | 0.10 | 2.26 | 0.01 | - | - | 0.001 | 0.9927 |
|  |  | 1.00 | 2.27 | 4.08 | - | - | 0.244 | 0.0284 |
|  | Among CAN concentrations | 1h | 3.33 | 5.45 | - | - | 0.333 | 0.0037 |
|  |  | 24h | 3.35 | 1.09 | - | - | 0.098 | 0.3658 |
|  |  | 24hR | 3.35 | 0.43 | - | - | 0.032 | 0.7346 |
| **Globulin** | Among sampling times | 0.00 | 2.21 | 4.78 | - | - | 0.312 | 0.0195 |
|  |  | 0.01 | 2.24 | 10.90 | - | - | 0.476 | 0.0004 |
|  |  | 0.10 | 2.22 | 5.84 | - | - | 0.346 | 0.0092 |
|  |  | 1.00 | 2.24 | 1.28 | - | - | 0.097 | 0.2971 |
|  | Among CAN concentrations | 1h | 3.30 | 3.18 | - | - | 0.243 | 0.0380 |
|  |  | 24h | 3.34 | 6.86 | - | - | 0.377 | 0.0010 |
|  |  | 24hR | 3.27 | 14.32 | - | - | 0.611 | <0.0001 |

**Table S3.** Summary of statistical analyses performed for Protein profile. Results are presented as the test statistic (F for ANOVA or H for Kruskal–Wallis), degrees of freedom (df), exact p-values, and effect sizes (η² for ANOVA and ε² for Kruskal–Wallis).

Tukey's test was used for multiple comparisons following significant ANOVA results, whereas Nemenyi's test was used following significant Kruskal-Wallis results. A dash (-) indicates that the statistic was not applicable.
