# Supplementary material for: Cellulose acetate nanoparticles: impacts of concentration and exposure time on the biochemistry and hematology of juvenile Colossoma macropomum
Source: Ecotoxicology. 2026 Oct 23;35(9):189. doi: 10.1007/s10646-026-03174-w (PMC13694297; doi:10.1007/s10646-026-03174-w)
Supplement: Supplementary file 4 [file 10646_2026_3174_MOESM4_ESM.docx]

| **Variable** | **Comparison** | **Condition** | **df** | **F** | **H** | **ε^2^** | **η^2^** | **p-value** |
| --- | --- | --- | --- | --- | --- | --- | --- | --- |
| Hemoglobin | Among sampling times | 0.00 | 2 | - | 7.12 | 0.254 | - | 0.0284 |
|  |  | 0.01 | 2 | - | 0.43 | 0.226 | - | 0.8048 |
|  |  | 0.10 | 2 | - | 2.81 | 0.100 | - | 0.2449 |
|  |  | 1.00 | 2 | - | 1.56 | 0.054 | - | 0.4592 |
|  | Among CAN concentrations | 1h | 3 | - | 11.09 | 0.284 | - | 0.0112 |
|  |  | 24h | 3 | - | 4.30 | 0.113 | - | 0.2304 |
|  |  | 24hR | 3 | - | 2.97 | 0.078 | - | 0.3960 |
| Hematocrit | Among sampling times | 0.00 | 2 | - | 4.15 | 0.154 | - | 0.1155 |
|  |  | 0.01 | 2 | - | 7.33 | 0.262 | - | 0.0235 |
|  |  | 0.10 | 2 | - | 2.63 | 0.101 | - | 0.2540 |
|  |  | 1.00 | 2 | - | 4.33 | 0.149 | - | 0.1063 |
|  | Among CAN concentrations | 1h | 3 | - | 0.87 | 0.023 | - | 0.8272 |
|  |  | 24h | 3 | - | 4.99 | 0.139 | - | 0.1619 |
|  |  | 24hR | 3 | - | 5.12 | 0.138 | - | 0.1517 |
| Erythrocytes | Among sampling times | 0.00 | 2.27 | 1.11 | - | - | 0.074 | 0.3440 |
|  |  | 0.01 | 2.26 | 0.05 | - | - | 0.003 | 0.9507 |
|  |  | 0.10 | 2.26 | 0.75 | - | - | 0.055 | 0.4820 |
|  |  | 1.00 | 2.7 | 1.26 | - | - | 0.086 | 0.3008 |
|  | Among CAN concentrations | 1h | 3.34 | 0.39 | - | - | 0.034 | 0.7625 |
|  |  | 24h | 3.34 | 0.71 | - | - | 0.056 | 0.5530 |
|  |  | 24hR | 3.36 | 0.94 | - | - | 0.074 | 0.4309 |
| MCV | Among sampling times | 0.00 | 2 | - | 0.88 | 0.037 | - | 0.6427 |
|  |  | 0.01 | 2 | - | 0.17 | 0.007 | - | 0.9183 |
|  |  | 0.10 | 2 | - | 1.11 | 0.046 | - | 0.5744 |
|  |  | 1.00 | 2 | - | 1.10 | 0.061 | - | 0.4968 |
|  | Among CAN concentrations | 1h | 3 | - | 3.45 | 0.099 | - | 0.3267 |
|  |  | 24h | 3 | - | 0.43 | 0.013 | - | 0.9340 |
|  |  | 24hR | 3 | - | 0.94 | 0.026 | - | 0.8151 |
| MCH | Among sampling times | 0.00 | 2 | - | 0.13 | 0.005 | - | 0.9348 |
|  |  | 0.01 | 2 | - | 1.18 | 0.047 | - | 0.5537 |
|  |  | 0.10 | 2 | - | 1.91 | 0.073 | - | 0.3857 |
|  |  | 1.00 | 2 | - | 1.72 | 0.061 | - | 0.4240 |
|  | Among CAN concentrations | 1h | 3 | - | 2.22 | 0.067 | - | 0.5290 |
|  |  | 24h | 3 | - | 0.32 | 0.009 | - | 0.9558 |
|  |  | 24hR | 3 | - | 3.70 | 0.100 | - | 0.2963 |
| MCHC | Among sampling times | 0.00 | 2.25 | 2.12 | - | - | 0.145 | 0.1412 |
|  |  | 0.01 | 2.24 | 0.76 | - | - | 0.060 | 0.4777 |
|  |  | 0.10 | 2.24 | 0.01 | - | - | 0.001 | 0.9907 |
|  |  | 1.00 | 2.27 | 0.22 | - | - | 0.016 | 0.8019 |
|  | Among CAN concentrations | 1h | 3.34 | 3.15 | - | - | 0.218 | 0.0374 |
|  |  | 24h | 3.33 | 2.65 | - | - | 0.194 | 0.0648 |
|  |  | 24hR | 3.33 | 2.65 | - | - | 0.194 | 0.0650 |

**Table S4.** Summary of statistical analyses performed for Hematological parameters. Results are presented as the test statistic (F for ANOVA or H for Kruskal–Wallis), degrees of freedom (df), exact p-values, and effect sizes (η² for ANOVA and ε² for Kruskal–Wallis).

Tukey's test was used for multiple comparisons following significant ANOVA results, whereas Nemenyi's test was used following significant Kruskal-Wallis results. A dash (-) indicates that the statistic was not applicable.
